# Perception and barriers to access Pre-exposure Prophylaxis for HIV/AIDS (PrEP) among the MSM (men who have sex with men) Brazilian Amazon: A qualitative study

**DOI:** 10.1371/journal.pone.0296201

**Published:** 2024-09-26

**Authors:** Diego Rafael Batista, Rafaela Nunes Dávila, Alicia Cacau dos Santos, Felipe Queiroz Rocha, Jessica Albuquerque Araújo, Aline Côrte Alencar, Loren Rebeca Nascimento, Nilberto Dias de Araújo, Stefanie Costa Pinto Lopes, Patricia Saraiva Araújo, Rondienny Andrade Filgueiras, Priscila Ferreira Saraiva, Marcus Vinicius Guimarães de Lacerda, Djane Clarys Baía-da-Silva, Felipe Leão Gomes Murta

**Affiliations:** 1 Universidade do Estado do Amazonas, Manaus, Brazil; 2 Fundação de Medicina Tropical Doutor Heitor Vieira Dourado, Manaus, Brazil; 3 Fundação Hospitalar de Hematologia e Hemoterapia do Amazonas, Manaus, Brazil; 4 Instituto Leônidas & Maria Deane, Fiocruz, Manaus, Brazil; 5 University of Texas Medical Branch, Galveston, Texas, United States of America; 6 Universidade Nilton Lins, Manaus, Brazil; Ruedi Luethy Foundation - Zimbabwe, ZIMBABWE

## Abstract

Pre-exposure prophylaxis (PrEP) is an effective HIV prevention strategy that consists in the use of antiretroviral drugs by seronegative people at risk of HIV. Negative perceptions, inadequate understanding, and access barriers have been associated with decreased medication adherence. Manaus is the largest city in the Brazilian Amazon, where the incidence of HIV/AIDS is high, and the rates of adherence to the antiretroviral treatment for HIV and PrEP are low. In this qualitative study among PrEP users, mostly MSM, we explored perceptions, knowledge, and access barriers. We conducted 21 in-depth interviews with an intentionally sampled group of participants who had used PrEP at least once in their lifetime, selected through the snowball technique, between April and July 2022. A thematic analysis was conducted with a predominantly inductive approach. We highlight three relevant themes: (i) access to information about PrEP and its influences on users, (ii) access, monitoring, and barriers encountered, and (iii) facilitators for PrEP adherence and sexual behaviors. One of the negative perceptions identified in the study involves a misunderstanding of the association between PrEP users and the HIV/AIDS status. Participants revealed that some non-PrEP users suspect that individuals claiming PrEP usage are concealing an HIV-positive status to engage in unprotected sex. Lack of information by health professionals regarding HIV prevention methods poses significant barriers to PrEP access and adherence. Participants emphasized social media’s crucial role in PrEP awareness. The results suggest a need to increase digital outreach regarding PrEP, decentralize PrEP services, and provide comprehensive healthcare training to improve the effectiveness of the preventive measure.

## Introduction

HIV infection still is a significant global public health challenge, disproportionately affecting vulnerable populations in low- and middle-income countries [1–3]. In 2022, there were 39.0 [33.1–45.7] million people living with HIV, of whom 86% [73–>98%] knew their HIV status [4]. New HIV infections have decreased by 59% since the peak in 1995, and this is due to expanded testing, pre-exposure prophylaxis (PrEP), HIV post-exposure prophylaxis (PEP), treatment of people living with HIV, reducing viral load, and consequently promoting undetectable status [4–8]. However, these measures must be better implemented to ensure the objectives of the United Nations and World Health Organization program to end the AIDS epidemic and reduce transmission, morbidity and mortality, associated costs, and social stigma by 2030 [9]. Within this context, PrEP poses an effective approach to preventing HIV transmission that involves the use of medications (TDF and FTC) by individuals who are not infected with the virus but are at risk, such as men who have sex with men (MSM), transgender individuals, sex workers and partners with mixed HIV status [10–16]. This strategy aims to be proactive, administering medications before any potential exposure to the virus occurs [14, 15].

PrEP, under the conditions of clinical trials and in real-life settings, significantly reduces the risk of HIV infections by more than 90% and 93%, respectively [17–20]. Furthermore, it has relevant impacts in several areas. First, it contributes to reducing anxiety related to sex and the risk of HIV infection. Second, it promotes greater liberty and pursuit of users in health services to manage the risk of contracting HIV. This includes the regular search for guidance on prevention, vaccination, testing, and treatment for sexually transmitted infections. Additionally, it enables autonomous sexual decision-making, regardless of sexual activity or partner approval [21–23]. Although having important and with significantly positive results and impacts, medication adherence rates are still low and can be explained by race/ethnicity disparities, persistence of safe sex status, mainly due to relationships with steady and long-lasting partners, sexual behavior, difficulty with services offering PrEP, low knowledge and awareness, low perception of HIV risk, fear of stigma, concern about adverse and side effects, burden related to daily medication use, language barriers, prejudice by service providers, among others [24–27]. These barriers and obstacles to accessing and adhering to PrEP highlight a critical vulnerability to HIV infection, underscoring the need for these factors to be recognized and addressed through the development of more effective interventions [28, 29]. Building on this understanding, different studies have aimed to explore the perceptions, knowledge, limitations, and facilitators on PrEP access among at-risk populations. The findings reveal a complex array of facilitators and barriers that are influenced by cultural, social, behavioral, and sexual factors. This diversity emphasizes the importance of tailoring interventions to meet the unique needs and circumstances of each group, ensuring more equitable and effective access to PrEP across different communities [30, 31].

Since 2017 in Brazil, PrEP has been incorporated into the Unified Health System (SUS), a national health system that guarantees universal and free health coverage for all residents, covering everything from preventive care to complex medical procedures. Which means, PrEP has been available since then at no cost in various health units throughout the country [32]. Despite advances in the provision of PrEP, some barriers still limit access for potential users, such as limitations to the inadequacy of the service in the context of users’ lives and work, especially the most vulnerable; stigma and discrimination related to gender identity; poverty, racism, gender inequalities and criminalization of sex work [33]. These barriers were evaluated in the five regions of the country, including Manaus. However, due to Brazilian regional variations in health care services and multicultural aspects of the population, such barriers, and facilitators of access to PrEP must be highlighted by region.

The Brazilian Amazon is a vast territory, characterized by its diversity and cultural complexity. It is home to a mix of indigenous peoples, riverside communities, and migrants. These groups live in varied socioeconomic and cultural conditions, often facing a scarcity of resources and health services. This situation impacts their quality of life and access to health care [34–36]. The combination of these human and environmental factors makes the Amazon region in Brazil one of the most affected by infectious diseases, including HIV [37, 38]. To illustrate this, the incidence of HIV/AIDS in the Brazilian Amazon is high (26.8%), and the rates of adherence to antiretroviral treatment for HIV and PrEP are low (50% and 4.9%, respectively) [39–41]. Manaus, for example, is the largest city in the Brazilian Amazon and a significant urban center. It is the second Brazilian city with the highest number of people living with HIV/AIDS (18.5 thousand cases) and deaths (261 in 2022) and has low indicators of health and access to health services, characteristic of the northern region [36, 39, 40]. In Manaus, PrEP started being provided as part of the project for the Implementation of the Pre-Exposure Prophylaxis (ImPrEP) and PrEP Brazil studies. These studies are focused on evaluating how effectively PrEP can be delivered to populations at risk of HIV infection, its acceptance among these groups, and the overall impact on HIV prevention efforts [20, 42]. Initially introduced in 2016, the program expanded in 2018, decentralizing to additional Primary Health Care Units (PHCU) in Manaus to broaden access [43]. The most recent data from the local government in 2022 revealed that a mere 600 individuals across the city were consistently utilizing PrEP [44]. Nevertheless, since 2018, around 3,700 people had engaged with PrEP at some juncture. Given the elevated HIV incidence and the city’s population exceeding 2 million, the accessibility of PrEP remains confined to selected healthcare facilities [45]. For this reason, we conducted a qualitative study in Manaus, using in-depth interviews with individuals who have used the PrEP service at least once to investigate their perceptions, knowledge, and barriers to HIV PrEP.

## Materials and methods

### Study design and site description

This study adopted a descriptive, exploratory qualitative approach, using in-depth interviews (IDIs) to describe the various factors influencing access and adherence to HIV PrEP in a Brazilian Amazon context.

**Fig 1:**
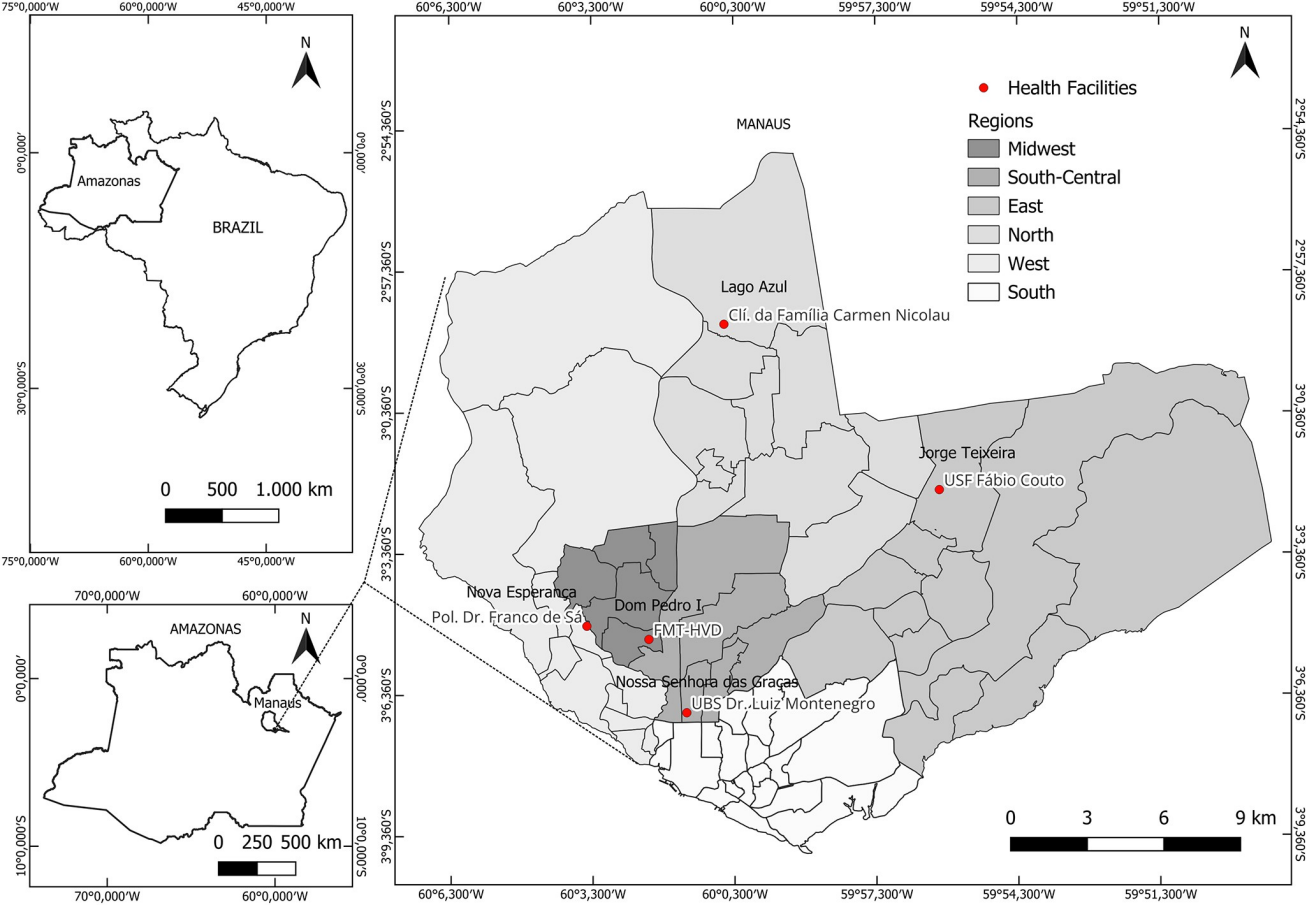
Geographic distribution of PrEP service centers in Manaus during the study period. The base used to create the map is from the IBGE (Brazilian Institute of Geography and S, which is freely accessible for creative uses in shapefile format, in accordance with the Brazilian Access to Information Law (12,527/2011) (https://geoftp.ibge.gov.br/cartas_e_mapas/bases_cartograficas_continuas/bc250/versao2019/).

The study was carried out in Manaus, the capital of the state of Amazonas, a Brazilian city with the highest number of HIV cases reported in recent years [40]. In the city, PrEP has been offered free of charge by the Unified Health Service (SUS) since 2018, and it’s currently available in 5 units in the urban area. One operating within as the reference hospital in the state of Amazonas for HIV/AIDS, another concurrently with the specialized care, operates in a polyclinic and the other are 3 primary health care units, out of the 288 existing in the city (Fig 1).

### Participant selection and recruitment

Participants were recruited using the linear snowball technique, a method chosen due to the sensitive nature of the research topic and the challenges associated with accessing this specific group of individuals in the city [46]. The research topics’ inherent privacy and personal nature required a participant observation method that provided access to this population. The two initials participants were recruited based on their previous interactions with the research team at an HIV referral hospital. They belonged to a specific social cluster, MSM, with a higher education level. Consequently, they had social contact with other PrEP users. Utilizing the snowball sampling method, each of these initial participants recommended another PrEP user from their social contacts, who were then approached by the study team for participation. The first contact with all participants happened by phone, followed by a face-to-face explanation of the research.

### Qualitative data collection

A semi-structured interview guide was developed and validated through a preliminary process with a smaller sample of five PrEP users who did not participate in the main study. This validation process involved conducting trial interviews with these individuals to assess the clarity, relevance, and comprehensiveness of the questions in the guide. Feedback from these participants was then used to refine and adjust the interview questions to better capture the experiences and perspectives relevant to PrEP use, ensuring the guide’s effectiveness for the larger study. Among the issues addressed in the questions were the perceived effectiveness of PrEP, barriers accessing the medication, adherence to treatment, and participants’ experiences with medical follow-up related to PrEP (Table 1). Discussion topics and questions were refined from discussions and agreements between two experts in HIV and Psychology.

**Table 1:** Overview of the validated interview guide.

Questions	Objective
***Perception about PrEP***	
How did you find out about PrEP? Can you share your experience?	Describe the knowledge and intention to use PrEP among vulnerable populations and potential users in Manaus. We investigated what participants know about PrEP, their sources of information, and their motivations for seeking and using the HIV prevention method.
***PrEP impact***	
What is your opinion about PrEP (ease of use, safety, etc)? Could you tell me what you think about the reliability of PrEP?	Understand the perceived impact of pre-exposure prophylaxis (PrEP) use on users’ lives, including possible behavioral changes regarding HIV prevention. Among the questions investigated are adherence to HIV testing, the combined use of other prevention tools, and whether the use of PrEP influenced participants to adopt behaviors that may put them at greater risk of HIV infection. In addition, we explored whether PrEP use impacted other aspects of participants’ lives, such as quality of life and mental health.
***Challenges for adherence to PrEP***	
Can you describe what made it easier and what barriers did you find, or do you face in continuing to use PrEP? What do you think would lead a person to stop using PrEP?	Comprehend the barriers to accessing PrEP and the factors that facilitate its use to provide information that can improve the implementation and promotion of PrEP in the region.

In-depth interviews were conducted between April 5, 2022, and July 18, 2022, on an intentional sample of participants who used PrEP at least once in their lifetime. The interviews were conducted by DRB, who had previous experience with qualitative data collection, in a private, safe, and quiet room to maintain anonymity and avoid excessive noise and discomfort. The IDIs lasted an average of 45 minutes and audios were later transcribed (LN, NDA) in full without personal identifiers. The number of interviews was determined by the principle of theoretical saturation where IDIs are carried out until a clear pattern appears and subsequent groups do not produce new information. One couple was interviewed together at the request of the interviewees, generating a unique audio file. Transcripts were reviewed by members of the research team (DRB, RND, ACS and FR) to correct any transcription errors and ensure the accuracy of participants’ responses.

### Data analysis

The IDIs’ transcripts (S1 Text) were imported into the MAXQDA20 program. Members of the research team (DRB, PSA, RAF, and PFS) independently developed a codebook (S2 Text) and performed line-by-line coding of the interviews. Subsequently, a thematic analysis was conducted, applying a predominantly inductive approach [47], and the creation of categories after having previously read the transcripts that emerged during the analysis process. After that, we conducted thorough discussions to explore and refine the identified themes and emerging subthemes. Any disparities that surfaced during the coding process were effectively resolved through consensus discussions.

### Research team and reflexivity

The study team consists of five PhD qualitative researchers, two female researchers (DCBS, SCPL) and three male researchers (FLGM, NDA, MVGL), with expertise studies focused on infectious diseases. The study team is also comprised of six master’s students (DRB, RND, FR, FQR, JAA, RAF, PFS, PSA), two doctoral students (ACS, LRN), and a physician (ACA), all with consolidated experience with HIV in Amazonian populations.

FLGM, MVGL, and ACS had previously conducted qualitative research with vulnerable population in the Amazon region [48, 49]. The team members made efforts to ensure that their subjectivity did not influence the collection and analysis of data and the study team had no prior relationship with the participants.

### Ethical approval and participant consent

This study was approved by the Research Ethics Committee of the Fundação de Medicina Tropical Doutor Heitor Vieira Dourado, CAAE: 49882721.7.0000.0005. All participants provided written informed consent before participating in the study. The confidentiality of all participants was maintained at all stages of the research, data was recorded anonymously, and voluntary participation was documented on the Informed Consent Terms. All consolidated criteria for reporting qualitative research (COREQ) were followed in the production of this manuscript to ensure a high-quality report (S3 Text) [50].

## Results

### Characteristics of the interviewees

Twenty-one participants were interviewed, and the average age was 28 [standard deviation (SD) = 4.75]. Most participants live in the South-central Zone of Manaus, and most units that offer PrEP are often distant from these participants. Participants were mainly cis men and MSM (n = 19) and had completed higher education (n = 15). Due to the method of snowball sampling and the challenges in including more stigmatized social groups in Brazil, which are less represented in PrEP services—such as transgender women and Indigenous peoples—the participant pool was predominantly limited to these demographics. This limitation reflects the broader issue of access and inclusion within health services, highlighting the need for targeted outreach and support strategies to ensure all at-risk communities can benefit from PrEP. At the time of the interviews, three were not using PrEP. The period used varied between one month and four years (Table 2).

**Table 2:** Characteristics of the population studied.

ID	Age	PrEP status	Education (years)	Sexual orientation	Occupation	Health unit offering PrEP	Approximate time (in years) of PrEP use
01	34	Discontinued	11	MSM	Party promoter	PHCU/ THU	3,5
02	32	In use	≥ 15	MSM	Healthcare professional	PHCU	3
03	24	In use	≥ 15	bisexual	Advertising professional	PHCU	2
04	25	In use	≥ 15	MSM	Public relations	THU	2
05	22	Discontinued	11	bisexual	Waiter	THU / PHCU	2
06	27	In use	≥ 15	MSM	Veterinarian	THU	4
07	35	In use	≥ 15	MSM	Teacher	THU	4
08	27	In use	≥ 15	MSM	Healthcare professional	PHCU	0.08 (1 month)
09	34	In use	≥ 15	MSM	Veterinary	PHCU	1
10	37	In use	≥ 15	MSM	Physical educator	PHCU	3,5
11	24	In use	11	MSM	Healthcare professional	PHCU	0.08 (1 month)
12	23	In use	11	MSM	Healthcare professional	PHCU	3 (interruptus)
13	31	In use	≥ 15	MSM	Public server	PHCU	2
14	35	In use	≥ 15	MSM	social worker	THU	2
15	24	In use	≥ 15	MSM	Journalist	THU/PHCU	3
16	25	In use	≥ 15	MSM	Healthcare professional	PHCU	4
17	25	In use	11	MSM	Store manager	PHCU	1
18	35	In use	≥ 15	MSM	Civil engineer	THU	4
19	24	In use	≥ 15	MSM	Advertising professional	THU	4
20	27	Discontinued	≥ 15	MSM	Advertising professional	THU	3
21	25	In use	11	MSM	Real estate	THU	4

PHCU = Primary Health Care Units; THU = Tertiary Health Unit.

### Thematic analysis

The thematic analysis resulted in three major themes to categorize the reports: Theme 1: "Access to information about PrEP and influences on use"; Theme 2: "Access, monitoring and barriers encountered"; and Theme 3: "Facilitators for adherence to PrEP and sexual behaviors."

#### Theme 1: Access to information about PrEP and influences on its use

This theme covers how participants are familiar with and understand PrEP. Some participants reported that their first information about PrEP was predominantly acquired through social media and influencer profiles. Participants also highlighted obtaining and encouraging the search for knowledge about PrEP within the circle of friends, loved ones, and sexual partners.

*“[…] A friend shared about PrEP on his Instagram story*, *which made me curious*, *and I started searching the subject to find out more.”*

(Participant 10, MSM, 37 yrs old).

*“[*…*] I found out about PrEP on Instagram*. *Some pages promoting this content caught my attention and I went to investigate information on websites”*

(Participant 13, MSM, 31 yrs old).

The fear of HIV infection and the need for ideal sexual safety and freedom were factors that motivated the search for PrEP. Raising awareness by an HIV-positive partner within a serodifferent relationship was also a stimulus for seeking and adhering to PrEP.

*“I went out with a boy*. *I went to his house*. *We hooked up*. *At his house*, *he introduced me to PrEP and explained it to me*. *I told him I didn’t know it and hadn’t even used it*. *I immediately accepted the information provided*, *for me*, *prevention is always super important and any type of protection I accept and pursue”*

(Participant 05, bisexual, 22 yrs old).

*“[*…*] My choice to use PreP was due to fear of HIV infection*. *We see in the newspapers that the state of Amazonas is in first place in the ranking of infected people*. *So*, *the decision to use PrEP was motivated by fear [*…*]”*

(Participant 06, MSM, 27 yrs old).

*“[*…*] I wore a condom*, *but I was afraid of breaking it and sometimes wearing a condom during sexual intercourse wasn’t so cool*. *I was always afraid of HIV*

(Participant 16, MSM, 25 yrs old).

*“I had a relationship with an HIV-positive partner*, *[*…*] it was through him that I discovered PrEP*. *He said*, *"as I’m positive*, *I prefer that you do the treatment so that we can prevent ourselves" so it was a way of trying to prevent myself*…*"*

(Participant 11, MSM, 24 yrs old).

The use of PEP, an emergency HIV prevention strategy, was cited to include some interviewees in PrEP. Awareness of the possibility of exposure to HIV, associated with some sexual practices and the free medication, led some to start the prophylaxis.

*“[…] I was in a relationship with a steady partner*, *but he had been in a relationship with someone else for two months (laughs)*. *He and I were having sex without a condom*, *I was only with him and whether I like it or not*, *I was insecure*. *Insecurity led me to want to use PrEP.”*

(Participant 07, MSM, 35 yrs old).

*“[*…*] I always talk about the percentage of protection that PrEP gives the user*. *Knowing about PrEP was one thing that encouraged me to use PrEP*, *because I have an active sex life and PrEP is free*, *which means a great thing to me”*

(Participant 04, MSM, 25 yrs old).

#### Theme 2: Access, monitoring, and barriers encountered

This theme encompasses some of the main implications related to access and continuity or discontinuation in the PrEP program, according to the participants’ perception. Two subthemes were addressed: subtheme 01 “Long distances and inconvenient opening hours for PrEP access” and subtheme 02 “Prejudice and stigma”.

*Subtheme 01*: *Long distances and inconvenient opening hours for PrEP access*. The distance between the health unit that offers PrEP and the participant’s residence, in addition to the scarcity of health units that offer PrEP for a large metropolis like Manaus, the operation of health units only during business hours in addition to precarious transport conditions in the city were considered a barrier to the use of PrEP and demotivator to maintain its use.

*“[…] The fact that PrEP [services] only work in the morning is a negative point for those who work*. *Service could be more flexible”*

(Participant 04, MSM, 25 yrs old).

*“[*…*] my ex-husband [*…*] used PrEP*. *He did it for a month and abandoned it because he found the health unit to be too far away [*…*] he gave up due to the difficulty in going to the place where he was taking PrEP [*…*]”*

(Participant 07, MSM, 35 yrs old).

*“[*…*] if I could schedule PrEP follow-up on the weekends*, *for me*, *it would help a lot [*…*] I was unable to start using PrEP again because follow-up visits always take place on weekdays*, *and my work now is at the same time*, *I can’t even take time off”*

(Participant 19, MSM, 24 yrs old).

*Subtheme 02*: *Prejudice and stigma*. According to participants, there is a wrong association between PrEP use and HIV/AIDS status in the general population. For these people, HIV-positive people use PrEP to hide their HIV status, which results in prejudice and stigma towards PrEP users.

*“In the minds of some*, *like my father and my aunt*, *I was not taking PrEP*, *but ART*, *because my husband was HIV positive*, *and I was hiding my serology”*

(Participant 01, MSM, 34 yrs old).

*“[*…*] there are people who look at the packaging of the medication (PrEP) and confuse it with ART [*…*] I*, *myself*, *at the beginning of PrEP*, *hid the pots and didn’t leave them on the dresser because I was embarrassed [*…*]”*

(Participant 05, bisexual, 22 yrs old).

Participants using PrEP are pejoratively considered “*promiscuous*,” careless, or assumed to take part in *“sexual orgies*.*”*

*“[…] I’ve been told that people use PrEP to have sexual orgies […]”*

(Participant 07, MSM, 35 yrs old).

*“[*…*] many of my friends don’t even know about PrEP*, *and they think the reason I’m taking the medication is that I want to have sex without a condom*!*”*

(Participant 08, MSM, 27 yrs old).

*“[*…*] what I hear the most is that people who take it are 100% having sex without a condom*, *without care and trusting PrEP 100% [*…*]”*

(Participant 19, MSM, 24 yrs old).

#### Theme 3: Facilitators for PrEP adherence and sexual behaviors

Feelings of security, freedom, and confidence in relation to sexual health were evidenced regarding the use of PrEP. Another benefit was the improvement in users’ mental health.

*“[…] I feel more protected if something happens […] maybe if I were in a monogamous relationship*, *I could even take off the condom during relations”*

(Participant 02, MSM, 32 yrs old).

*“[*…*] PrEP has helped with my mental health*, *my sexuality… I feel more at ease*. *[*…*] I feel more protected from paranoia and outbursts*, *whereas before if I thought I had something*, *or that I exaggerated*… *I would get upset and not be able to go to college*. *I just thought about that because I have anxiety”*

(Participant 07, MSM, 35 yrs old).

*“[*…*] it’s living with the anxiety that consumed me*. *Before PrEP*, *I still tried a lot to wear condoms*, *… I’d slip back*, *get excited and stop using it and it was always the same thing*: *Paranoia*, *guilt and then rushing to take a quick test*.*”*

(Participant 18, MSM, 35 yrs old).

After being linked to PrEP, the interviewees could fully access the health system, as previously there were negative relationships. Thus transforming the general healthcare routine into a habit. The offer of regular testing and immunizations through vaccines in addition to the constant presence of physiological assessments and laboratory tests, were reported by participants as positive factors for adherence to PrEP.

*“Before PrEP*, *I went for a quick test […] the health professional in the service*, *[…] he must have had the best of intentions… He made me aware of the importance of wearing condoms*, *but he said: ‘you wouldn’t be here if you had used a condom’*. *With the stress I was dealing with*, *the anxiety I was feeling and the paranoia I had created*, *the impact was very negative”*

(Participant 18, MSM, 35 yrs old).

*“After PrEP [*…*] I end up knowing what is happening in my body because of the medication [*…*] there is a list of blood tests that we do*, *such as vitamin D levels*. *It is done to find out if the PrEP medication is causing any side effects”*

(Participant 04, MSM, 25 yrs old).

All participants understood that the medication used in PrEP only offers protection against HIV. After PrEP adhering, there was an increase in routine screening for STIs. Participants reported using other methods to prevent STIs, in addition to PrEP.

*“[…] I happened to have a relationship with a cis man*, *and he didn’t want to wear a condom: ‘oh*, *but you take PrEP! Don’t you take PrEP?’*, *[…] It’s not just HIV*, *we are vulnerable to several other infections […]”*

(Participant 03, bisexual, 24 yrs old).

*“[*…*] I was tested for STIs*, *but not very often*, *but since PrEP I have been tested regularly”*

(Participant 08, MSM, 27 yrs old).

*“[*…*] many people before PrEP were not in the habit of being tested for STIs*. *With PrEP*, *you are encouraged to take exams even every 3 months*, *but it is a window smaller than once a year*. *So*, *I think it was a big difference*, *and for me it’s just a benefit*. *[*…*] a friend who is also on PrEP discovered that he had an STI in the middle of the process*. *He wouldn’t find that out if they weren’t using PrEP and doing regular testing"*

(Participant 18, MSM, 35 yrs old).

*"[…] we have several ways of combining prevention*: *testing*, *lubricating gel*, *condoms*, *getting to know who you interact with [SEROLOGICAL STATUS] […] during the follow-up we learned about other forms of prevention*, *which we didn’t know about until then*, *such as knowing that lubricating gel is not just an item to make sexual intercourse more pleasurable [*…*]"*

(Participant 01, MSM, 34 yrs old).

There are reports of a reduction in the frequency and/or number of sexual partners stimulated by self-care in sexual relations. Despite some participants mentioning not wearing condoms in most sexual relations. they highlighted that this is not directly related to PrEP but it’s an already established behavior. They also described that they worry about other STIs but feel good knowing that they are protected against HIV.

*“[…] I think that with the use of PrEP my sex life decreased*, *it became more regulated because I knew more about the care*. *Nowadays*, *I have an active sex life*, *[‥] because I think I… worry more actually”*

(Participant 04, MSM, 25 yrs old).

*“[*…*] I recognize that I have a risky sexual behavior*, *I am a gay man*, *I have sex with other men*, *and I don’t usually wear condoms*. *I don’t do well with condoms*. *I understand the need and importance*, *but I don’t get used to it*. *I do not like it*. *I think I perform poorly during sex*. *Therefore*, *I recognize that STI is a very big concern*. *However*, *PrEP helps eliminate my biggest concern [HIV]”*

(Participant 18, MSM, 35 yrs old).

*“[*…*]… it’s not that I’m going to stop wearing condoms*. *But in some situations*, *like oral sex*, *I believe I won’t wear it [*…*] with medication I feel safer having oral sex without a condom*.*”*

(Participant 10, MSM 37 yrs old).

Table 3 shows the main challenges and solutions related to PrEP access in Manaus, as identified through interviews. It addresses issues such as the difficulty in accessing health units, inconvenient opening hours, discrimination, and a lack of information about PrEP. Proposed solutions include expanding the distribution of PrEP service throughout more health care units, applying mobile health services, extending operating hours, training healthcare staff in inclusivity, and enhancing PrEP awareness through social media campaigns.

**Table 3:** Barriers and facilitators to PrEP access identified by interviewed participants, predominantly MSM, in Manaus.

Barriers	Details	Proposed Facilitador/Solutions
**Distance and lack of transportation**	Long distances to PHCUs and inadequate transportation options make accessing PrEP challenging for many participants.	Develop a strategy for the wider distribution of PHCUs offering PrEP. Implement mobile health units equipped for PrEP delivery.
**Inconvenient PrEP operating hours**	PHCUs operating only during traditional business hours limit access for working individuals.	Extend PHCU operating hours to include early mornings, evenings, and weekends.
**Prejudice and stigma**	Misunderstanding and stigma associated with PrEP use, including misconceptions that PrEP users are HIV-positive.	Comprehensive training for healthcare professionals on cultural competency and inclusivity. Public awareness campaigns to educate on the benefits and use of PrEP.
**Lack of awareness**	Insufficient promotion and knowledge of PrEP within the community.	Launch public awareness campaigns across various media platforms. Increase digital outreach and social media presence.
**Healthcare professionals bias**	Discomfort and prejudice from healthcare providers when discussing PrEP and HIV risk behaviors.	Training programs for healthcare professionals to improve health communication and reduce bias. Create supportive environments for discussing HIV prevention openly.

## Discussion

PrEP represents a significant advance in HIV/AIDS prevention, but there are multiple perceptions surrounding this intervention, as well as multiple barriers and difficulties in use, adherence, and awareness [51]. This multiplicity may be related to socioeconomic, cultural, gender, and sexual behavior [52]. For the first time, to our knowledge, we evaluated, through a qualitative study focusing on the experiences of MSM, perceptions, knowledge, and barriers to HIV PrEP in patients treated outside of clinical trials in the important Brazilian Amazon city.

Based on the evaluation of interview responses, our study showed that social networks play a central and significant role in mediating information about PrEP and reaching potential users. Other studies have already demonstrated social networks’ importance in interventions or health knowledge dissemination [53–60]. Using social media to disseminate a disease prevention strategy enables target audience engagement. It can increase the visibility of specific health issues, making them more interesting and acceptable, especially for the young population [61–63]. Information about PrEP available on the internet allows potential users free access to information in the field of sexual health, especially for adolescents who may feel uncomfortable expressing their doubts and needs in the family/community context [64, 65]. Considering that in Brazil, more than 50% of HIV detection cases occurred among young people aged 20 to 34 [40], digital platforms should be considered in the strategic planning of health education actions to promote HIV prevention through PrEP by authorities, mainly when targeted at younger populations or those with low education [66–68]. However, it is important to highlight that, despite considerable progress in expanding internet access in Brazil, there are still populations that do not have this connection, especially in the Amazon region [69].

The main factors identified as stimulating the search for PrEP were the simplicity of the method, the perception of reduction risk associated with sexual practices, and the feeling of freedom in the exercise of sexuality with the possibility of safer sexual relations, even without the use of a condom. Another crucial factor that encouraged engagement and continuity to PrEP was the reduction in feelings of stress and anxiety associated with unprotected sexual intercourse. Other studies show perceptions and facilitators for PrEP like those we highlighted, although they evaluated different regions and populations across different countries [70–73]. One study showed that among PrEP-eligible men in Australia, PrEP use was independently associated with lower levels of anxiety about HIV and suggested that this finding may help promote, increase demand for, and adhere to PrEP [74]. Emphasizing the positive “side effects” of PrEP on mental health, according to Price et al. [75] could be a strategy to target priority populations for HIV prevention. By alleviating anxiety and stress, PrEP can help reduce costs associated with treating mental disorders, as well as increase the insertion of populations vulnerable to HIV into the labour market and improve academic and professional qualification, once mental health problems are currently essential factors regarding absence from work or school, reduced productive capacity, among others [76, 77].

The interviewees also pointed out access to comprehensive healthcare as an important perception. They mentioned having facilities for rapid HIV testing and testing for other infectious diseases, in addition to examinations to assess their overall health conditions, all available within the health unit. These services extend beyond those recommended in clinical PrEP protocols [78]. Also, the feeling of sexual freedom associated with PrEP is a positive perception regarding this preventive therapy, being, according to other authors, a facilitator for seeking, adhering to, and engaging with the health service [79–82]. Given the feeling of freedom expressed, we showed that the participants have knowledge about combined prevention.

The geographic distance from the interviewees’ residence to the health units that offer PrEP and transportation difficulties were identified as significant obstacles in accessing the service. Even among participants who had a car for transportation, as traffic in the city is intense, especially while PrEP health units are open, corroborating previous studies [33, 70]. Therapeutic itinerary studies in search of treatment or prevention of HIV [83–86] are already available for several regions, but no studies have been carried out for Amazonia, and studies of this nature need to be carried out and should serve as a tool to guide government actions for better access to PrEP and HIV treatment. In Manaus, the combination of physical distance and the scarcity of prophylaxis access points was mentioned as one of the possible reasons for involuntary or voluntary interruption of PrEP use. This reflects a significant barrier to access to the service, like that observed in other regions and HIV-related services [87, 88].

The underwhelming reception by health professionals, coupled with their lack of information about the forms of HIV prevention available in the health system, was cited as a significant barrier to PrEP access and adherence. This barrier is associated with health professionals’ discomfort in discussing HIV risk behaviors with patients, their resistance to prescribing it, their own prejudice, and it’s especially related to sexual prejudice [89–91].

Another critical barrier reported in our study concerns prejudice and stigma related to the general population’s misunderstanding of the distinction between PrEP users and people living with HIV/AIDS, with those who use PrEP being wrongly labeled as individuals with HIV/AIDS. This perception is strongly associated with the prejudice that still exists against people living with HIV in Manaus and could be demystified with the use of communication tools mentioned by participants, such as PrEP posts on social media [92, 93].

The study had important limitations regarding sample diversity, it did not include any trans women due to the difficulty of approaching this population in Manaus. Consequently, the study primarily focused on participants who are cis men and MSM. Difficulties in recruiting other demographic groups, including adolescent girls, young women, women, black individuals, and indigenous people, stemmed from the stigma surrounding HIV/AIDS and restricted availability of PrEP for these communities in Manaus. Additionally, the impossibility of the presence of an observer during the IDIs, due to the sensitivity of the topic, may have impacted the objectivity and scope of the observations. The qualitative nature of the study may also restrict the extent of conclusions, as individual perceptions and experiences may not fully reflect the diversity of perspectives that exist in the target population.

## Conclusion

In conclusion, we highlight, for the first time, the perceptions and barriers to adherence to PrEP in the Brazilian Amazon region. Our participants, predominantly MSM, demonstrate positive perceptions and knowledge about PrEP, emphasizing the importance of social networks in disseminating information. Significant barriers to PrEP access and adherence include long geographical distances to health units offering PrEP, lack of reception by health professionals, and a deficiency of information about HIV prevention methods available in the healthcare system. The results suggest the need for greater dissemination of PrEP information through digital media, decentralization of PrEP services to additional health units across the city, and comprehensive training of health teams to provide adequate and inclusive prophylaxis, particularly for the most vulnerable populations.

## Supporting information

S1 Text Codebook used in the qualitative analysis of interviews for the study. (DOCX)

S2 Text Consolidated criteria for reporting qualitative research (COREQ). (DOCX)

S3 Text Consolidated criteria for reporting qualitative studies (COREQ): 32-item checklist. (DOCX)
