# Supplementary material for: Perception and barriers to access Pre-exposure Prophylaxis for HIV/AIDS (PrEP) among the MSM (men who have sex with men) Brazilian Amazon: A qualitative study
Source: PLoS One. 2024 Sep 26;19(9):e0296201. doi: 10.1371/journal.pone.0296201 (PMC11426485; doi:10.1371/journal.pone.0296201)
Supplement: S1 Text — (DOCX) [file pone.0296201.s001.docx]

**Codebook in Portuguese**


Qualiprep.mx20


24/01/2023

**Lista de Códigos**

| 1 Divulgação | 18 |
| --- | --- |
| 2 Apoio no circulo social | 19 |
| 3 PrEP e Saúde mental | 44 |
| 4 PEP | 8 |
| 5 entendimento sobre HIV/AIDS | 23 |
| 6 Importância social da PrEP | 45 |
| 7 dúvidas | 23 |
| 8 Entendimento de Outras ISTs | 19 |
| 8.1 rastreio | 26 |
| 9 Regularidade da prep/uso | 72 |
| 10 PrEP injetável | 40 |
| 11 Avaliação da tecnologia | 1 |
| 11.1 Abandono de PrEP | 59 |
| 11.2 Mudança de hábitos | 11 |
| 11.3 Acompanhamento da Prep | 106 |
| 11.4 Segurança e confiança | 84 |
| 11.5 Efeitos adverso | 37 |
| 11.6 Entendimento sobre PrEP | 75 |
| 11.7 Facilidades | 28 |
| 11.7.1 atendimento humanizado | 15 |
| 11.7.2 profissionais capacitados | 9 |
| 11.7.3 facilidade de acesso | 57 |
| 11.7.3.1 dispensação e farmácia | 23 |
| 11.8 preconceito e estigma | 105 |
| 11.8.1 Recusa de atendimnento | 5 |
| 11.9 acesso | 41 |
| 11.10 Dificuldades | 61 |
| 11.10.1 profissionais despreparados | 13 |
| 11.10.2 Dificuldade de acesso | 94 |
| 11.10.3 Falta de informação | 39 |
| 11.11 Tempo de uso de PrEP | 22 |
| 12 Outros metodos de prevenção utilizados | 40 |
| 13 Comportamento sexual | 58 |
| 14 Melhorias no serviço de PrEP | 135 |
| 15 Estímulo ao uso de PrEP | 66 |
| 16 Apresentação a PrEP | 37 |

**1 Divulgação**

**2 Apoio no circulo social**

**3 PrEP e Saúde mental**

**4 PEP**

**5 entendimento sobre HIV/AIDS**

**6 Importância social da PrEP**

**7 dúvidas**

**8 Entendimento de Outras ISTs**

**8.1 Entendimento de Outras ISTs >> rastreio**

**9 Regularidade da prep/uso**

**10 PrEP injetável**

**11 Avaliação da tecnologia**

**11.1 Avaliação da tecnologia >> Abandono de PrEP**

**11.2 Avaliação da tecnologia >> Mudança de hábitos**

**11.3 Avaliação da tecnologia >> Acompanhamento da Prep**

**11.4 Avaliação da tecnologia >> Segurança e confiança**

**11.5 Avaliação da tecnologia >> Efeitos adverso**

**11.6 Avaliação da tecnologia >> Entendimento sobre PrEP**

**11.7 Avaliação da tecnologia >> Facilidades**

**11.7.1 Avaliação da tecnologia >> Facilidades >> atendimento humanizado**

**11.7.2 Avaliação da tecnologia >> Facilidades >> profissionais capacitados**

**11.7.3 Avaliação da tecnologia >> Facilidades >> facilidade de acesso**

**11.7.3.1 Avaliação da tecnologia >> Facilidades >> facilidade de acesso >> dispensação e farmácia**

**11.8 Avaliação da tecnologia >> preconceito e estigma**

**11.8.1 Avaliação da tecnologia >> preconceito e estigma >> Recusa de atendimnento**

**11.9 Avaliação da tecnologia >> acesso**

**11.10 Avaliação da tecnologia >> Dificuldades**

**11.10.1 Avaliação da tecnologia >> Dificuldades >> profissionais despreparados**

**11.10.2 Avaliação da tecnologia >> Dificuldades >> Dificuldade de acesso**

**11.10.3 Avaliação da tecnologia >> Dificuldades >> Falta de informação**

**11.11 Avaliação da tecnologia >> Tempo de uso de PrEP**

**12 Outros metodos de prevenção utilizados**

**13 Comportamento sexual**

**14 Melhorias no serviço de PrEP**

**15 Estímulo ao uso de PrEP**

O que levou o participante buscar e iniciar o uso

**16 Apresentação a PrEP**

De que maneira a pessoa conheceu a PrEp
