# Supplementary material for: Perception and barriers to access Pre-exposure Prophylaxis for HIV/AIDS (PrEP) among the MSM (men who have sex with men) Brazilian Amazon: A qualitative study
Source: PLoS One. 2024 Sep 26;19(9):e0296201. doi: 10.1371/journal.pone.0296201 (PMC11426485; doi:10.1371/journal.pone.0296201)
Supplement: S2 Text — (DOCX) [file pone.0296201.s002.docx]

**Consolidated criteria for reporting qualitative studies (COREQ): 32-item checklist - Perception and Barriers to access Pre-exposure Prophylaxis for HIV/AIDS (PrEP) in the Brazilian Amazon: a qualitative study**

Developed from:

Tong A, Sainsbury P, Craig J. Consolidated criteria for reporting qualitative research (COREQ): a 32-item checklist for interviews and focus groups. *International Journal for Quality in Health Care*. 2007. Volume 19, Number 6: pp. 349 – 357

| **No. Item** | **Guide questions/description** | **Reported on Page #** |
| --- | --- | --- |
| **Domain 1: Research team and reﬂexivity** |  |  |
| *Personal Characteristics* |  |  |
| 1. Inter viewer/facilitator | *Which author/s conducted the interview or focus group?*  DRB | Pg 8. |
| 2. Credentials | *What were the researchers’ credentials?*  PhD, MD, BSc, MSc, MRes, | Pg 8. |
| 3. Occupation | *What was their occupation at the time of the study?*  Physicians, master students, PhD students, and qualitative researchers. | Pg 8. |
| 4. Gender | *Was the researcher male or female?*  Some researchers were male, others were female. | Pg 8. |
| 5. Experience and training | *What experience or training did the researcher have?*  Renowned researchers, as well as graduate students with experience in qualitative research, all with qualitative research training and with published articles in the area. | Pg 8-9. |
| *Relationship with participants* |  |  |
| 6. Relationship established | *Was a relationship established prior to study commencement?*  None of the participants had an established relationship with an author prior to study commencement. | Pg 9. |
| 7. Participant knowledge of the interviewer | *What did the participants know about the researcher? (e.g., personal goals, reasons for doing the research).*  The initial two participants were recruited based on previous interactions with the research team in an HIV referral hospital. | Pg 6. |
| 8. Interviewer characteristics | *What characteristics were reported about the inter viewer/facilitator? e.g., bias, assumptions, reasons and interests in the research topic.*  No interviewer-related biases were identified. | Pg 8. |
| **Domain 2: study design** |  |  |
| *Theoretical framework* |  |  |
| 9. Methodological orientation and Theory | *What methodological orientation was stated to underpin the study? e.g., grounded theory, discourse analysis, ethnography, phenomenology, content analysis.*  Members of the research team (DRB, PSA, RAF, and PFS) performed independently a thematic analysis with a predominantly inductive approach. | Pg 8. |
| *Participant selection* |  |  |
| 10. Sampling | *How were participants selected? e.g., purposive, convenience, consecutive, snowball.*  Snowball | Pg 6. |
| 11. Method of approach | *How were participants approached? e.g., face-to-face, telephone, mail, email.*  The first contact with all participants was by phone, following a face-to-face explanation of the research. | Pg 6. |
| 12. Sample size | *How many participants were in the study?*  21 PrEP users | Pg 9. |
| 13. Non-participation | *How many people refused to participate or dropped out? Reasons?*  No one has given up on participating in the project. | N/A |
| *Setting* |  |  |
| 14. Setting of data collection | *Where was the data collected? e.g., home, clinic, workplace.*  The interviews were conducted by DRB, who had previous experience with qualitative data collection, in a private, safe, and quiet room to maintain anonymity and avoid excessive noise and discomfort | Pg 8. |
| 15. Presence of non-participants | *Was anyone else present besides the participants and researchers?*  No, only the interviewer was in the room during the interview. | Pg 8. |
| 16. Description of sample | *What are the important characteristics of the sample? e.g. demographic data, date*  These data are described in the study results | Pg 9. |
| *Data collection* |  |  |
| 17. Interview guide | *Were questions, prompts, guides provided by the authors? Was it pilot tested?*  The questions were developed by Research Team and previously tested and validated by the researchers in a smaller sample of volunteers and adjustments were made to ensure the transparency and relevance of the questions. | Pg 6. |
| 18. Repeat interviews | *Were repeat inter views carried out? If yes, how many?*  No | N/A. |
| 19. Audio/visual recording | *Did the research use audio or visual recording to collect the data?*  The interviews were recorded and transcribed without personal identifiers, so that the database could be anonymized | Pg 8. |
| 20. Field notes | *Were ﬁeld notes made during and/or after the inter view or focus group?*  No | Pg 21. |
| 21. Duration | *What was the duration of the interviews or focus group?*  The interviews lasted an average of 45 minutes | Pg 8. |
| 22. Data saturation | *Was data saturation discussed?*  The number of interviews was determined by the principle of theoretical saturation where SIs are carried out until a clear pattern appears and subsequent groups do not produce new information | Pg 8. |
| 23. Transcripts returned | *Were transcripts returned to participants for comment and/or correction?*  No. | N/A. |
| **Domain 3: analysis and ﬁndings** |  |  |
| *Data analysis* |  |  |
| 24. Number of data coders | *How many data coders coded the data?*  Four researchers independently developed a codebook and performed line-by-line coding. | Pg 8. |
| 25. Description of the coding tree | *Did authors provide a description of the coding tree?*  No. | N/A |
| 26. Derivation of themes | *Were themes identiﬁed in advance or* *derived from the data?*  The analysis of the interviews and the field notes allowed us to identify three major themes. | Pg 12. |
| 27. Software | *What software, if applicable, was used to manage the data?*  MAXQDA 20 program | Pg 8. |
| 28. Participant checking | *Did participants provide feedback on the ﬁndings?*  No. | N/A |
| *Reporting* |  |  |
| 29. Quotations presented | *Were participant quotations presented to illustrate the themes/ﬁndings? Was each quotation identiﬁed? e.g. participant number*  Yes, quotations were presented to illustrate the themes/findings, and each quotation was identified with a participant number. | Pg 12-18. |
| 30. Data and ﬁndings consistent | *Was there consistency between the data presented and the ﬁndings?*  Yes, there was consistency between the data presented and the findings. | Pg 12-18. |
| 31. Clarity of major themes | *Were major themes clearly presented in the ﬁndings?*  Yes, major themes were clearly presented in the Results section using specific sections regarding each theme. | Pg 12-18. |
| 32. Clarity of minor themes | *Is there a description of diverse cases or discussion of minor themes?*  Yes, minor themes were discussed. | Pg 12-18. |
